# Neurodivergence and Threat: A Case Study on the Risk of Violent Fixations in Autism Spectrum Disorder

**DOI:** 10.7759/cureus.76379

**Published:** 2024-12-25

**Authors:** Thayjas A Patil, Syed Y Qadri, Daisy V Shirk

**Affiliations:** 1 Psychiatry, Drexel University College of Medicine, Philadelphia, USA; 2 Psychiatry, Reading Hospital Psychiatry Residency, Reading, USA; 3 Child and Adolescent Inpatient Unit, Tower Behavioral Health, Reading, USA

**Keywords:** autism spectrum disorder (asd), extremism, fixation, mass shooting, mental health, pediatric, perseveration, risk assessment, risk of violence

## Abstract

Mass shootings have increasingly captured public attention in recent decades, prompting closer examination of the mental health of those responsible. This scrutiny often focuses on individuals with neurodevelopmental disorders, such as autism spectrum disorder (ASD). While epidemiological evidence is mixed on whether these individuals are more likely to commit acts of violence than the general public, certain behavioral characteristics may make them more vulnerable to extremist ideations. This case study focuses on a 17-year-old male patient who initially presented with suicidal behavior, later diagnosed with ASD following clinical evaluation. He was also found to have additional comorbidities such as major depressive disorder, gender dysphoria, and unspecified eating disorder, while harboring threats of extreme violence. Despite displaying characteristics similar to previous mass shooters with ASD, the patient has never acted on his violent thoughts, a pattern consistent with most individuals with violent ideations. The case overviews the complexity of assessing when such threats are legitimate and the potential consequences of misinterpretation. There is urgent need for standardized protocols to differentiate between behaviors stemming from ASD and violence unrelated to ASD. The findings highlight the importance of understanding the vulnerabilities and presentations of neurodivergent individuals to provide appropriate care and prevent potential tragedies.

## Introduction

As mass shootings have increased, media reports have consistently drawn attention to the mental health of those responsible. This has not only raised public concern about the connection between mental health and violence but also increased negative stereotyping of neurodivergent individuals [1]. It is estimated that the prevalence of autism spectrum disorder (ASD) among children in the United States is approximately 2.76% [2]. According to the Diagnostic and Statistical Manual of Mental Disorders, fifth edition (DSM-5), ASD is characterized by persistent deficits in social communication and interaction across multiple contexts, along with restricted and repetitive patterns of behavior, interests, or activities. Symptoms must be present in the early developmental period, although they may not fully manifest until later. These symptoms must cause clinically significant impairments in social, occupational, or other important areas of functioning and cannot be better explained by intellectual disability or global developmental delay. A systematic review of people with ASD in the criminal justice system suggests that those with ASD are no more likely to commit extremism or common crimes than the general public [3]. In fact, it has been shown that ASD individuals across the United States and Canada are more likely to be the victims of a crime rather than offenders [4]. Conversely, a 2017 study analyzing U.S. mass shootings from the Mother Jones database identified evidence of ASD in six out of 75 cases (8%), with an additional 16 cases exhibiting evidence of ASD-like trait, a prevalence notably higher than that of the general population [5]. As more data emerges, it is important for clinicians to continue to delineate this phenomenon in order to understand it better.

Whether or not neurodivergent individuals are at increased risk of extremism, there are certain behavioral traits that can increase their susceptibility to extremist tendencies. These include circumscribed interests, need for consistency, social deficits, poor communication skills, and sensory sensitivities. Circumscribed interests, often seen in ASD and Asperger Syndrome, involve intense, focused, and repetitive interests that take on an obsession-like quality [6,7]. These interests are researched extensively for great periods of time, providing a sense of comfort. These individuals usually engage well with a high level of expertise when conversing about their topic of interest. Depression and anger can often intensify more negative interests as a way of processing those emotions [8]. The need for consistency and adherence to rules in ASD usually acts as a protective factor, as most ASD individuals are law-abiding. However, unpredictable situations and inconsistencies can cause distress as they struggle to make sense of or correct the situation [9]. Some of the most unpredictable situations ASD individuals face are social interactions due to difficulties perceiving social cues, nonverbal communication, and paralinguistic communication like tone. These deficits have the potential to make them take statements literally and cause distress when mimicked social scripts are ill perceived in the wrong context. These skills are usually less necessary in online forms of communication where conversations can be more scripted and rehearsed, leading ASD individuals to a more solitary lifestyle [10]. Sensory sensitivities may reinforce this withdrawal, making new experiences overwhelming, while hyposensitivities can lead them to seek more compelling stimuli, such as certain types of information or visuals online. Although evidence on sensory sensitivities is limited, they are potential vulnerabilities that should be considered [11].

Many of these vulnerabilities are on display in our case of a 17-year-old male patient presenting for suicide attempt, while displaying signs of major depressive disorder, ASD, gender dysphoria, and unspecified eating disorder, later found to be harboring threats of extreme violence.

## Case presentation

The patient is a 17-year-old male adult admitted to an inpatient psychiatric facility in Pennsylvania following two overdoses of acetaminophen and ibuprofen. He was adopted by his paternal grandparents at age six and has lived with them since. He was reported to be an excellent student, having completed his education online. While described as reserved and isolative, he occasionally socialized with peers during weekly aviation group meetings. He had no prior medical or psychiatric diagnoses or history of mental health treatment.

On initial evaluation, the patient appeared guarded, hesitant to answer questions. He exhibited limited eye contact, difficulty interpreting social cues, and spoke with restricted inflection. He denied symptoms of depression, anxiety, psychosis, or other mood disorders, and he denied substance use. The patient stated he had “blanked out” prior to the suicide attempt and did not remember what happened.

In the following days of hospitalization, the patient started to open up and disclosed depression symptoms of depressed mood, anhedonia, guilt, hopelessness, decreased energy and concentration, and frequent thoughts of not wanting to be alive. He revealed feeling “different” from his peers, without understanding why, and reported difficulty communicating with his adoptive parents. He endorsed distress related to his gender identity, stating that he did not feel comfortable in his own body, yet still expressing preference for the pronouns he/him. He admitted to restricting food intake to achieve the “ideal” body he saw in women's magazines. He stated that he had overdosed because he felt he did not “belong” in this world and believed his adoptive parents would never understand him.

Based on clinical encounters and collateral information, he was diagnosed with major depressive disorder, gender dysphoria, an unspecified eating disorder, and ASD. The ASD diagnosis was based on his social communication difficulties, challenges forming relationships, limited eye contact, focus on routines, and concrete thinking. He was informed that neuropsychological testing would be necessary for a definitive diagnosis. While initially distressed by the ASD diagnosis, he later found comfort in having a plausible explanation for his thought process. He was started on escitalopram for depressive symptoms and aripiprazole for disordered thinking.

During the hospitalization, the treatment team spoke extensively with the patient’s adoptive parents. They explained that despite their best efforts to support him, the patient consistently expressed a “natural disdain” toward them, especially toward the adoptive mother. They stated that they would accept him “no matter what” but expressed concern after presenting disturbing text messages they discovered, where he discussed his desires to kill them and fantasies about self-harm. They also found a video of him dancing with a gun, which they had thought was securely locked. On his computer, they discovered search queries such as “school shooter police response times” and recipes for pipe bombs. When questioned, the patient offered seemingly calculated explanations for these actions, downplaying them as mere “curiosity.” He later admitted that prior to hospitalization, he had thoughts of “punishing” lawmakers who target lesbian, gay, bisexual, transgender, queer, questioning, intersex, asexual all other sexual and gender identities (LGBTQIA+) youth. He then rationalized that committing an act of mass violence would have the “opposite of the intended effect”. He was subsequently started on aripiprazole for disordered thinking. He categorically denied homicidal ideation throughout hospitalization.

On the unit, the patient exhibited impulsive and erratic behavior, including headbanging, punching walls, and poor boundaries with peers. He made an alarming comment to peers, saying, “Make Columbine Great Again,” referencing the Columbine school shooting, upsetting several individuals in the milieu. He later stated it was “a joke in poor taste.” Following this, he wrote a two-page letter expressing suicidal thoughts and was subsequently found attempting to use a sheet to hang himself in the bathroom to “fix the problem”. Weeks later, the patient tried to elope from the unit. He kicked open a door, made a large hole in the wall, and needed to be sent to the emergency room to get stiches. He tried attacking nursing staff and made homicidal threats toward the psychiatrist.

Given the significant evidence of dangerous behavior, the patient’s adoptive parents expressed concern for their safety and stated they were uncomfortable with him returning home. Members of the treatment team also expressed concern about the potential threat of mass violence. The team, in consultation with administration, notified the police and Children and Youth Services (CYS) of the patient’s threats and that the adoptive parents had been advised to remove all weapons from the home. An independent forensic psychiatrist was also consulted to complete a threat assessment. A final recommendation was made to transfer the patient to a residential treatment facility, given the numerous predictors of violence (Table 1).

**Table 1:** Patient ASD Characteristics and Predictors of Violence ASD: autism spectrum disorder; LGBTQIA+:  Lesbian, Gay, Bisexual, Transgender, Queer/Questioning, Intersex, Asexual, and all other sexual and gender identities

ASD Characteristics	Predictors of Violence
Circumscribed Interests	Video dancing with guns; Searches for school shootings, police response times, and pipe bomb recipes; Homicidal threats in text messages
Difficulty in Communication and Interpreting Social Cues	Comments like “Make Columbine great again,” upsetting peers; Poor boundaries
Concrete Thinking	Homicidal threats; Rationalization that committing violence would have the "opposite of the intended effect"
Social Withdrawal	Withdrawn and isolative; Suicidal behavior
Need for Consistency and Adherence to Rules	Wanted to “punish” lawmakers for targeting LGBTQIA+; Suicide attempt to “fix the problem”
Emotional Dysregulation	Headbanging; Punching walls; Kicking door; Physical aggression

## Discussion

In 2023, Weisbrot et al. looked at the characteristics of students who make threats. Among 157 school-referred youths from 19 school districts across eastern Long Island, 80% were referred for making a verbal threat and 29.3% brought a weapon to school. Most were boys (88.5%) and white (79.7%), with high incidences of bullying (43.4%), family trauma (52.6%), physical abuse (5.1%), sexual abuse (5.7%), and verbal abuse (36.3%). ASD or pervasive developmental disorder not otherwise specified, learning disorder, attention-deficit hyperactivity disorder (ADHD), any depressive disorder, oppositional defiant disorder, and anxiety disorder were frequently comorbid [12]. A 2016 review article examining all published studies on autism in relation to crime and violence identified 27 case reports involving individuals with ASD and acts of violence, including physical assault, sexual assault, arson, murder, and stalking or violent threats. Features of ASD thought to increase the likelihood of violence included impaired understanding of others' mental states, impaired nonverbal cue interpretation, restricted interests, and psychiatric comorbidities. Nevertheless, an increase of ASD in the criminal justice system overall was not found [13]. A more recent 2024 review article looking at the bi-directional link between ASD or autistic traits and criminal behavior continues to find a lack of definitive correlation [14].

Two examples of ASD in school shootings specifically can be found in recounts of the children involved in the shootings at Sandy Hook Elementary and Columbine High School. According to his father, the Sandy Hook shooter showed classic features of Asperger’s Syndrome such as poor eye contact, communication difficulties, relationship problems, narrowed interests, and preservation of sameness. Growing up, the child became increasingly withdrawn and isolated. He had major deficits in empathy for others and preferred online communication. Particularly hostile toward females, he wrote, “why females are inherently selfish” on his computer. He was fascinated with guns, taking photographs with a gun to his head, and he often frequented the shooting range. He displayed negative thought patterns, anxiety, and a sense of hopelessness [15]. The Columbine shooter exhibited pathologic narcissistic personality disorder with anti-social and borderline features, in addition to ASD-like features. Feeling like a non-conformist, he wanted to punish people for a long list of rejections and criticisms he had experienced, real or imagined. Gaining a superiority complex, a sense of injustice, and hatred of humanity over time, he often rehearsed the massacre in the period leading up to it, without ever expressing remorse for his actions or thoughts [16].

While ASD may increase vulnerabilities to extremism, these vulnerabilities can also create behaviors that resemble extremism but potentially mislead practitioners. For example, narrowed interests, repetition, and hyperfocus are often seen in ASD, but if the focus is related to terrorism, the research, accumulation of data, and manufacturing of items can be a result of the compulsion/obsession rather than true ideological commitment. However, there is still the potential for such obsessions to lead the individual to act on their compulsions [8].

This case presents a challenge in determining whether the patient's threats and thoughts of violence represent a manifestation of ASD. While the patient exhibits characteristics commonly associated with previous school shooters with ASD, such as concrete thinking, feelings of marginalization, impulsivity, and curiosity about violence, he has not committed acts of violence toward individuals other than nursing staff. Our clinical decisions hinge on whether these behaviors suggest a credible threat to others in his life.

The treatment team expressed varying levels of concern, ranging from minimal to highly serious, with perceptions influenced by each member's previous experience and their therapeutic relationship with the patient. It is also important to recognize the potential impact of the heightened media focus on mass shootings in shaping our response. In the absence of standardized protocols for such cases, we took precautionary measures by notifying CYS and local law enforcement and sought an independent threat assessment to mitigate potential bias.

Misinterpreting ASD-related behaviors as non-ASD-related behaviors could lead to significantly different treatment approaches, potentially causing severe disruption to the patient, impacting the patient's opportunities for a normal life. Similarly, improper treatment could lead to devastating consequences, so these concerns must never be dismissed. If extremist ideations are driven by curiosity rather than ideological commitment, it may be possible to redirect these interests toward healthier outlets that provide similar relief from compulsions. Addressing underlying issues such as the need for intervention during perceived injustices and restoring predictability is crucial. In addition, treating comorbidities like depression and anxiety and improving social and communication skills is essential to prevent harmful behaviors.

## Conclusions

Individuals with neurodivergent disorders, such as ASD, exhibit vulnerabilities that have the potential to lead to thoughts or threats of harm, occasionally linked to mass violence. This case underscores the difficulty in determining whether these threats are related to ASD and the risks of misinterpretation. Standardized protocols and treatment channels are needed to efficiently address violence threats in ASD patients, ensuring proper identification and timely involvement of care and safety services. While it remains unclear if neurodivergent individuals are truly at a higher risk of extremism, it is imperative that clinicians understand their vulnerabilities in order to prevent the enormous distress capable of carrying out a tragedy.
